# A strategic initiative to facilitate knowledge translation research in rehabilitation

**DOI:** 10.1186/s12913-020-05772-8

**Published:** 2020-10-23

**Authors:** Katherine Montpetit-Tourangeau, Dahlia Kairy, Sara Ahmed, Dana Anaby, André Bussières, Marie-Ève Lamontagne, Annie Rochette, Keiko Shikako-Thomas, Aliki Thomas

**Affiliations:** 1 School of Rehabilitation, Faculty of Medicine, Université de Montréal, P.O. Box 6128, Station Centre-Ville, Montreal, Quebec H3C 3J7 Canada; 2 Centre for Interdisciplinary Research in Rehabilitation, Montreal, Quebec Canada; 3 School of Physical and Occupational Therapy, Faculty of Medicine and Health Sciences, McGill University, 3654 Sir William Osler, Montreal, Quebec H3G 1Y5 Canada; 4 Department of Epidemiology, Biostatistics, and Occupational Health, McGill University, 1020 Pine Avenue West, Montreal, Quebec H3A 1A2 Canada; 5 CanChild Centre for Childhood Disability Research, McMaster University, 1400 Main Street West, Room 408, Hamilton, Ontario L8S 1C7 Canada; 6 Département Chiropratique, Université du Québec à Trois-Rivières, 3351, boul. Des Forges, C. P. 500, Trois-Rivières, Quebec G9A 5H Canada; 7 Département de réadaptation, Université Laval, Quebec, Quebec G1V 0A6 Canada; 8 Center for Interdisciplinary Research in Rehabilitation and Social Integration, Institut de Réadaptation en Déficience Physique de Québec, 525 Boul Wilfrid-Hamel, Quebec, Quebec G1M 2S8 Canada; 9 Institute of Health Sciences Education, Faculty of Medicine and Health Sciences, McGill University, 1110 Pine Avenue West, Montreal, Quebec H3G 1A3 Canada

**Keywords:** Knowledge translation research, Strategic initiative, Implementation, Evidence-based practice, Physical rehabilitation, Physical disabilities

## Abstract

**Background:**

While there is a growing body of literature supporting clinical decision-making for rehabilitation professionals, suboptimal use of evidence-based practices in that field persists. A strategic initiative that ensures the relevance of the research and its implementation in the context of rehabilitation could 1) help improve the coordination of knowledge translation (KT) research and 2) enhance the delivery of evidence-based rehabilitation services offered to patients with physical disabilities. This paper describes the process and methods used to develop a KT strategic initiative aimed at building capacity and coordinating KT research in physical rehabilitation and its strategic plan; it also reports the initial applications of the strategic plan implementation.

**Methods:**

We used a 3-phase process consisting of an online environmental scan to identify the extent of KT research activities in physical rehabilitation in Quebec, Canada. Data from the environmental scan was used to develop a strategic plan that structures KT research in physical rehabilitation. Seven external KT experts in health science reviewed the strategic plan for consistency and applicability.

**Results:**

Sixty-four KT researchers were identified and classified according to the extent of their level of involvement in KT. Ninety-six research projects meeting eligibility criteria were funded by eight of the fourteen agencies and organizations searched. To address the identified gaps, a 5-year strategic plan was developed, containing a mission, a vision, four main goals, nine strategies and forty-two actions.

**Conclusion:**

Such initiatives can help guide researchers and relevant key stakeholders, to structure, organize and advance KT research in the field of rehabilitation. The strategies are being implemented progressively to meet the strategic initiative’s mission and ultimately enhance users’ rehabilitation services.

## Background

In different public health systems, there is a growing need to synthesise, adapt and apply the exponential amount of scientific evidence being generated in order to respond to patients’ expectations to receive the best possible care [1, 2]. Rehabilitation professionals, as vital members of interdisciplinary healthcare teams, offer services often in complex and unique practice settings aimed at enabling individuals with disabilities to reach and maintain their optimal physical, sensory, intellectual, psychological and social functional levels, and to optimize their participation in desired life domains [3]. Rehabilitation as a distinct field of practice often requires unique and tailored methods for knowledge translation (KT).

Despite a growing body of research in rehabilitation that can be used to support clinical decision-making and interventions to improve patient outcomes [4–10], studies have identified suboptimal research utilization in rehabilitation throughout the life span and across various conditions such as stroke [11], musculoskeletal disorders [12–20], paediatric conditions [21] and other chronic conditions [22, 23]. Barriers to uptake of research findings in rehabilitation include lack of time, limited confidence in the critical appraisal and use of research information, and limited support from management [17, 24–26]. Higher academic degrees, participation in research and close proximity between researchers and clinicians, student supervision and collaborative practice environments are factors found to facilitate research use [27–30]. Organizational determinants such as leadership style, social capital and the availability of resources [31–33] can also influence clinicians’ uptake of evidence-based practice (EBP). Studies conducted in rehabilitation suggest that systems-level changes and shifts in the organization’s paradigm, such as involving rehabilitation professionals in the research teams, can reduce the aforementioned barriers and promote a culture of EBP [27, 34–36].

Knowledge translation (KT) research in rehabilitation aims to identify individual and organisational barriers and facilitators and to develop, implement and assess the impact of strategies used to narrow the research-practice gap. Despite KT being a growing field, recent systematic reviews suggest that the amount and quality of the evidence on effective KT strategies to promote the use of EBP in rehabilitation is still limited [11, 37, 38]. The reviews also show a paucity of research on active, complex KT interventions as well as on the methods that may be used to evaluate the success of these KT interventions. Moreover, there is an absence of coordinated efforts aimed at developing and implementing KT plans and of training in that field [17, 39, 40]. As a KT plan is increasingly required by a growing number of funding agencies [41], researchers need to integrate effective KT strategies based on implementation science to build their KT plan [41], and include the relevant stakeholders throughout the research process [42].

Even though resources are limited, conducting KT research in rehabilitation using theory-based KT interventions and assessing robust outcome measures is needed [42]. Efforts should aim to increase the body of research in KT in rehabilitation and strengthen the implementation and evaluation of KT strategies in this context [43–45]. From a research standpoint, there is a need to collectively build the necessary bridges among people and organizations developing KT initiatives in clinical practice to share and improve the conduct of KT research [42]. Researchers and graduate students would benefit from having access to mentoring [46, 47]. Importantly, there is thus a need to support efforts to accelerate the uptake of EBP in clinical practice by advancing the KT research agenda in rehabilitation.

One way forward is to better understand the structures that can support researchers in designing, implementing, and evaluating KT strategies. This paper reports on the development and outcomes of the *Knowledge Translation Strategic Initiative in Rehabilitation in Quebec* (KT-SIRQ). This initiative leads, monitors and facilitates research developments in KT in rehabilitation for individuals with physical disabilities in one large Canadian province.

The objective of this paper is to describe a multiphase systematic process used to create the initiative and the initial applications of the implementation of the initiative’s strategic plan. We used the Knowledge-to-action (KTA) framework [48] to strategically address EBP gaps in rehabilitation in different settings.

## Methodology

### Context

This study took place in Quebec, Canada, where each of the ten provinces and  three territories has its own Ministry of Health and Social Services (MHSS), but the Canadian health care system is under the jurisdiction of the federal Ministry of Health. In Quebec, the MHSS administers health and social services across the province. A delegated minister is responsible for Rehabilitation, Youth Protection, Public Health and Healthy Living. Rehabilitation is a major priority across the country and most rehabilitation service structures are common between provinces [49–51].

The process of developing the 5-year strategic plan consisted of two phases: 1) an environmental scan (phase 1); and 2) the development of the strategic plan using a modified Delphi approach (phase 2A) and its validation (phase 2B). The data from the environmental scan were obtained from public websites and did not require ethics approval. The core team members were involved in the Delphi which did not require ethics approval. The validation process was exclusively for improvement purpose and did not constitute in a research process, thus it also did not require ethical approval. Ethical approval was obtained by the Centre for Interdisciplinary Research in Rehabilitation of Greater Montreal (CRIR-1169-0616) and institutional convenience of the targeted establishments from which we recruited participants for the survey conducted in the initial applications of the strategic plan.

#### Phase 1: environmental scan

Environmental scans consist of an exploratory review used to examine the state of a particular system to better understand its needs and context [52–54]. The purpose of our environmental scan conducted between November 2014 and October 2015 was to 1) identify KT researchers whose work was focused on the various stages and/or components of the KT process and research activities in the field of rehabilitation for patients living with physical disabilities in the province; and 2) to document the state of KT research related to physical disabilities.

### Search strategy

All searches for the environmental scan were conducted online (e.g., research centers websites) and/or by contacting funding agencies and organizations by email or phone. The search initially included rehabilitation researchers in all universities within the province. The keywords were defined based on the steps of the KTA framework [48] and included among others ‘dissemination’, ‘knowledge synthesis’ and ‘implementation’. The terms used to represent KT vary greatly, which explains the large number of keywords used. These words were searched and identified in the researchers’ profiles, and eligibility criteria applied (Table 1) in other relevant websites, both in French and English.

**Table 1:** Eligibility criteria for researchers and research projects related to KT

Type of data	Eligibility criteria
Researchers found online (universities, research centers and FRQS)	Inclusion criteria: Researcher’s description, publication or project include: 1) at least one term related to KT^a^ AND 2) has a field of work related to physical disabilities Exclusion criteria: 1) A person not eligible to receive funding 2) Rehabilitation conducted in the field of mental health 3) Research conducted in other unrelated fields of research 4) Retired researcher 5) No identification of their work in KT in their description
Projects funded by all funding agencies and organizations found online	Inclusion criteria: 1) Project’s description or title include at least one term related to KT^a^ 2) Description rely to KT 3) Field of work related to physical disabilities Exclusion criteria: 1) Rehabilitation conducted in the field of mental health 2) Research conducted in other unrelated fields of research

^a^Find full list of words searched in Additional file 1

#### Researchers

We consulted the websites of: 1) all eight universities with a rehabilitation department (occupational therapy, physical therapy, nursing, psychology, kinesiology, chiropractic, speech therapy and audiology) across the province of Quebec (Additional file 2); 2) seven major physical rehabilitation research centers serving various patient populations (Additional file 3); and 3) the major provincial research funding organizations. Researchers who met the eligibility criteria were identified (Table 1). We extracted the researcher’s health profession, research affiliation, credentials, contact information, research area/fields of interest, recent publications and the website information.

The research group engaged in a two-round validation process to identify the extent to which the identified researchers were involved in KT research. Using the information extracted from the public websites, each member (*n* = 8) independently classified the KT researchers into one of four ordinal categories describing the researcher’s level of involvement in KT. Category 1 included researchers working primarily in KT; most, if not all of their research projects were related to KT and were aimed at advancing KT research or implementation science. For example, a researcher that had formal training in KT (PhD, postdoctoral training in KT or courses in KT) doing research on KT science was placed into category 1. The second category included researchers doing research in KT as well as in another (other) domain(s). For example, a researcher in the second category would be involved in KT activities as well as in primarily rehabilitation-focused projects. The third category included researchers involved mainly in other domains of research but incorporating components of KT in their projects. For example, a researcher developing clinical practice guidelines in their main area of research would be in the third category. Researchers in the fourth category were excluded from the final environmental scan because they did not meet eligibility criteria (e.g., included key words in their description, but were doing KT research related to mental health). The categories were developed by our team and informed by the KTA framework [48]. All researchers integrating one or more components of the KTA framework were considered involved in KT research (categories one, two and three).

#### Funded research projects

We searched funding agencies’ and organizations’ websites for KT funded projects. We also contacted (by phone or email) key members of these agencies and organizations who were responsible for, or knowledgeable about, the KT projects to identify other projects that may not have been available on the websites. The search strategies for the funding agencies are presented in Additional file 4.

We collected the following information for all projects funded between 2005 and 2015: the organization or funding agency, the title, authors/researchers, target audience of the research (e.g., for clinicians, graduate students…), year, amount received and short summary. Projects started and/or funded prior to 2005 were excluded as KT research during that time period was not formally called “KT” in Canada (since publication of seminal KT work and mandate to include KT in project applications) [48].

#### Phase 2A: strategic planning

Based on the collective expertise of the research group members in KT research in rehabilitation and drawing on the preliminary results from the environmental scan (e.g., number of researchers involved in KT and percentage of KT projects funded by funding agencies) (Additional file 5), we developed a 5-year strategic plan to facilitate and support KT researchers in physical rehabilitation. Guided by Holt et al’s framework (2015) of strategic planning [55], we held six meetings and a two-day retreat (led by a group member (AB) with expertise in strategic planning) to: a) refine and approve the group’s mission, vision and goals; b) identify the strategies (how we will reach the objectives) and tactics/actions (what we will do and who will lead the intervention to implement the strategies) to achieve the goals, and metrics to assess the tactics/actions; c) plan for the tactics/actions execution; and d) establish a preliminary timeline for the implementation of the strategic plan. We used an iterative brainstorming approach to systematically generate strategies and related tactics/actions and elaborate the metrics and action plan. A consensus was then established to decide on the inclusion of activities in the strategic plan.

Following the series of team meetings, we used a 3-round modified Delphi method with our team members to obtain a consensus on the priority rankings for all strategies [56, 57]. The Delphi approach was used to facilitate collaborative work within the research team. Strategies were highly ranked if they could have a major impact on the mission of the KT-SIRQ. The first round consisted of each group member individually ranking the strategies by priority. The frequency of answers was calculated for every statement and a table was prepared with a summary of the findings from the first round, including a space for the second-round ranking. We engaged in a second-round seeking at least 80% consensus on all items. A third round was conducted via a face-to-face meeting in order to achieve final consensus.

For each strategy, individuals or organizations that could potentially be involved in leading the strategy were considered and proposed, and a timeframe and an estimation of the magnitude of required resources (e.g., budget, research personnel) needed for the execution of the corresponding tactics or actions were identified.

#### Phase 2B: expert consultation and review of the strategic plan

As suggested in the Holt and al’s framework [55], we consulted nine provincial and national experts (researchers and stakeholders (research advisors and coordinators)) in the field of KT to review the strategic plan for consistency and applicability.). The goal was to obtain feedback that would help ensure that the objectives and plan were aligned with current and future priorities in KT research in rehabilitation, and that the strategic plan was consistent and realistic. These experts from diverse settings (KT research groups (e.g., province-based subgroups of the *Strategies for Patient Oriented Research (SPOR) Unit*) and health related organizations (e.g., *National Institute of Excellence in Health and Social Services*) were researchers, managers and public health coordinators involved in KT. Experts were provided with an executive summary, a first complete version of the strategic plan, and a list of five open-ended questions (Additional file 6). Comments gathered through the consultation with the experts provided external validation of the strategic plan. All the comments were gathered in a single document, analyzed and addressed anonymously. The feedback was used to refine the strategic plan.

## Results

### Phase 1: environmental scan

#### Researchers

**Fig. 1:**
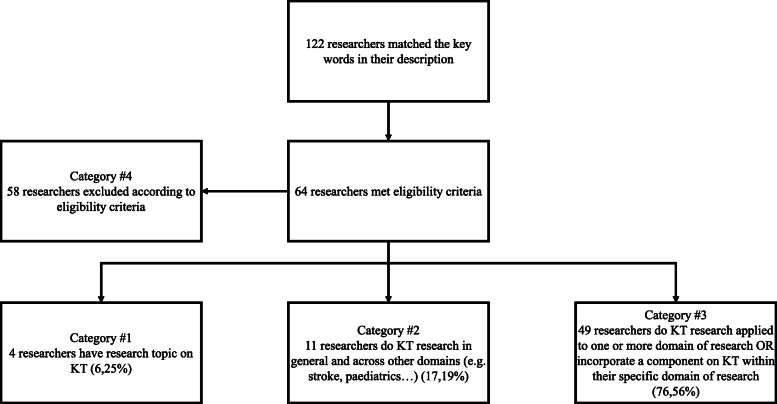
KT researchers selection flow diagram

We identified 123 researchers of which 64 matched the eligibility criteria after group consensus. The 64 researchers had a range of clinical and research training and were found across eight universities, seven research centers and one organization (i.e. *Fonds de recherche du Québec - FRQ*). Figure 1 illustrates the number and types of researchers that were identified and classified by the group members (four levels of classification – see description above).

#### Research projects

For the 10-year period between 2005 and 2015, a total of 96 projects funded by eight of the 14 different agencies met the eligibility criteria (Additional file 7). One provincial network in rehabilitation, funded a total of 14.4% of the projects related to KT in rehabilitation.

### Phase 2A: strategic planning

**Fig. 2:**
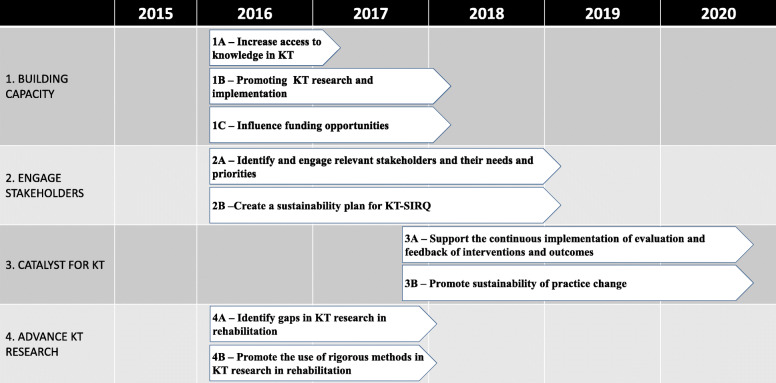
Five-year preliminary roadmap

The KT-SIRQ’s group mission, vision, four goals, nine related strategies (2–3 per goal) ranked by order of priority and 42 corresponding tactics or actions (3–10 per strategy) were elaborated. A 5-year preliminary timeline that underpins the strategic plan was also developed (Fig. 2). The strategic plan highlights the need to improve access to resources supporting the KT process; this would meet the need for guidance and structure in KT projects for researchers, students, postdoctoral fellows and stakeholders.

### Phase 2B: expert consultation and review of the strategic plan

Seven of the nine experts invited to review the strategic plan returned their feedback on whether the initiative aimed to build capacity in KT research or in KT more broadly; preliminary timelines for the proposed activities; the barriers and facilitators to the implementation of the strategic plan; the population targeted for each strategy; targeted leaders and a team for each strategy; and collaborations with existing initiatives in KT. We incorporated the comments and returned a revised version to group members for feedback and final approval. The final version of the strategic plan was completed after phases 2A and 2B (Additional file 8). Table 2 presents a summary of the strategic plan.

**Table 2:** Strategic plan summary

Strategic plan	
**Vision**: To enhance the health of individuals with physical disabilities in Quebec by advancing KT research in rehabilitation science. **Mission:** To build capacity and promote collaborative KT research in rehabilitation to improve the delivery of services and ultimately the health and wellbeing of individuals with physical disabilities.	
**Goals**	**Strategies**
1. Build capacity in KT research and implementation and promote networking	A. Increase access to knowledge in KT
	B. Promoting KT research and implementation
	C. Influence funding opportunities
2. Identify and engage relevant stakeholders (e.g., consumers of rehabilitation services, service providers, organizations, decision makers) to support the mission of the Qc KT Rehab Strat Initiative	A. Identify and engage relevant stakeholders and their needs and priorities
	B. Create a sustainability plan for KT-SIRQ
3. Be a catalyst for the creation, application and evaluation of innovative and effective KT for individuals with physical disabilities	A. Support the continuous implementation of evaluation and feedback of interventions and outcomes
	B. Promote sustainability of practice change
4. Advance KT research in rehabilitation	A. Identify gaps in KT research in rehabilitation
	B. Promote the use of rigorous methods in KT research in rehabilitation

### Moving forward from the strategic plan, preliminary outcomes

The strategies and actions elaborated in the strategic plan are being implemented according to the priorities established in the strategic plan, and each research group member is leading a strategy. The preliminary timeline was modified based on the funding and resources available for implementation of specific strategies. Our group also includes graduate students and post-doctoral fellows involved in ongoing projects. Three of the nine strategies have begun to be actively implemented by our group:

#### Strategy 1A: increase access to knowledge in KT

Consistent with our strategic plan and in collaboration with the federally funded KT provincial Component of the Canadian Institutes of Health Research (CIHR) SPOR Unit, we developed and held the first ever provincial KT training program to increase capacity in KT. The KT Component of the SPOR Unit aims to identify KT resources in healthcare and social services, and to support the creation and development of training programs in KT. Thirty-eight individuals (or researchers (*n* = 6), post-doctoral fellows (*n* = 7), graduate students (*n* = 13), master students (*n* = 4), research assistants (*n* = 2) and stakeholders such as clinicians, managers and research coordinators) from nine universities and seven clinical milieus participated in the first edition (2018) of the training program. The objectives of the 3-day training program were to: 1) Justify the importance of planning KT in research studies; 2) Identify and carry out effective and optimal KT strategies and methods in research projects and clinical projects including research on KT and implementation science; 3) Describe the steps to implement a KT plan; and 4) Create a KT plan for a grant application or a research project. Policy makers and patients were invited but were not represented.

#### Strategy 2A: identify and engage relevant stakeholders and their needs and priorities

Drawing from the environmental scan, we conducted a survey with identified researchers and stakeholders. Individual interviews and a focus groups with subsets of researchers working in rehabilitation are being held to identify needs and priorities in KT research. This ongoing study will identify the nature of their research, and the perceived barriers and facilitators to conducting KT research. Findings should help clarify both the priorities of individuals involved in KT across the province and their perceived need for support from our group.

Thirty-seven individuals (researchers (*n* = 33) and stakeholders (*n* = 4)) involved in KT research and/or activities in physical disabilities in the province completed the survey of the 94 invited to participate. In complementarity with the environmental scan, the survey revealed that researchers are primarily involved in KT activities in their main area of research and less than 20% are primarily focused on KT research. Though respondents were involved in various components of the KT process (e.g. knowledge syntheses, identification of EBP gaps and identification of barriers and facilitators to knowledge use) most were involved in knowledge syntheses (*n* = 22) and guidelines (*n* = 13), and the development of KT interventions (*n* = 16) rather than on evaluation (*n* = 12) or sustainability (*n* = 9) of KT strategies.

#### Strategy 4A: identify gaps in KT research in rehabilitation

In parallel with these two projects, we are currently in the process of conducting an overview of systematic reviews to answer the following research question. *In the context of health care, what is the impact of implementation strategies, when compared to none or other implementation strategies, to increase the application of evidence-based knowledge tools?* This synthesis will classify effective implementation strategies according to participants’ characteristics, health care domains and contexts to guide decision-making on the best ways to transfer evidence into clinical practice. Recommendations stemming from this review should enhance the use of KT methods and ultimately increase the quality of KT research in rehabilitation.

## Discussion

The purpose of this paper was to present the process and methods used to develop a strategic plan for an initiative that supports KT researchers in the rehabilitation of individuals with physical disabilities. This was accomplished through an environmental scan of KT researchers and research projects related to KT in the field and a consultation with key KT experts. We report on the first outcomes of the implementation of the strategic plan.

Based on the findings from the environmental scan, most of the researchers (76,6% of the researchers identified), were only partially involved in KT activities (category #3). Little emphasis from researchers seems to be made on advancing KT research or implementation science, defined as research aimed at improving methods to better use evidence in healthcare practices and policies (category #1, 6,2%) [58]. The same observation was made for the research projects as only very few of the research projects seem to incorporate one component of the KTA framework. Hence, it appears that researchers in physical rehabilitation seldom focus on the action cycle component of the KTA framework or employ integrated KT approaches as proposed by the Canadian Institute of Health Research (CIHR) [59]. Indeed, the majority of researchers in physical rehabilitation focus primarily on knowledge creation (e.g. development of practice guidelines) or end-of-grant KT projects with a dissemination plan. On their own, such projects are insufficient to affect outcomes on clinical practices [60]. Even though knowledge creation-type of research may not always lead to findings which can be immediately implemented in the clinical settings [61], additional attention should be given to the relevance of the research produced and to buy-in from relevant stakeholders to avoid widening the research-practice gap and increase the significance of research [59]. Integrated KT projects incorporating KT components early in the research process may increase with the growing emphasis on EBP in rehabilitation [62], stakeholder engagement in research [63], mounting pressure from local and federal funding agencies for researchers to include an implementation plan in grant proposals [59], and the increasing number of new investigators trained in KT [64]. For example, KT is part of the priority mandates for several major health funding agencies in in Canada (e.g., Canadian Institute of Health Research (CIHR) [59].

The large number of institutions and organizations interested in reducing research-practice gaps are other examples of the growing interest in KT and the importance of coordinating future KT research to optimize EBP. Previous research and this environmental scan further support the need to establish a structure that would facilitate and help advance KT research in rehabilitation [41]. By building capacity in KT research, identifying and engaging stakeholders, being a catalyst for KT and advancing KT research, our group will target essential aspects aiming to address sub-optimal use of robust methods in KT research. Using these strategies, and as research evidence in rehabilitation continues to grow, our aim is to facilitate and help coordinate developments in KT research. The structure proposed by our research group is an example of a strategic initiative that could support KT researchers and build capacity in KT research for ongoing and future studies. The macro-level strategic plan was developed using a rigorous process and suggests strategies that could be adapted to other health care research contexts in order to help build capacity in KT research. As recommended by the external KT experts who reviewed the strategic plan, the research group is working in collaboration with other initiatives in KT and stakeholder groups (patients, clinicians, healthcare managers, policy maker, and others) to implement the strategies (Bowen and Graham 2013), and promote the use of effective KT strategies in rehabilitation research, which is currently limited [37, 38]. This partnership with various actors involved in KT research could ultimately facilitate the use of EBP in clinical practice [65, 66].

Importantly, our findings suggest that a paradigm shift may be taking place in rehabilitation research. The results, combined with the strategic plan elaborated by the research group, highlight the need for a collaborative strategic endeavour to support the advancement and use of KT research and EBP in rehabilitation. The strategic plan developed in this study aims to structure and coordinate these advancements.

### Strengths and limitations of the process of creating a strategic initiative

To our knowledge, this project presents a first overview of the state of the research conducted since 2005 in KT in a large Canadian province. The environmental scan resulted in key information on researchers, research activities, and funding for KT research in physical rehabilitation. It is being followed with a survey to map the state of KT research in that province.

The process used to develop the strategic plan was rigorous and based on the literature and on the experience of one team member (AB) involved in previous strategic planning development. The mission, vision, goals, strategies and tactics/actions elaborated are in line with the gaps identified in KT research in the literature.

Nonetheless, our work has several limitations. According to a 2010 study by McKibbon and colleagues there are more than 100 terms used internationally to refer to KT [67]. Despite the large number of key words used in our environmental scan that we adapted from Graham’s work (2006) to search for KT researchers and projects, we may have missed resources and/or information. Many projects may contain a detailed implementation plan that was not described online, and therefore, may not have been captured in our search. In fact, systematic documentation of KT plans in research projects is, to our knowledge, not yet available. Further, our findings are based on information available online, from key people within the organizations contacted and from the collective expertise of the research group. We did not directly speak to the researchers identified. While the aim was not to highlight the precise nature and quality of available KT research in rehabilitation, this would be a worthwhile endeavour to advance the field. This scan focused on KT research targeting a specific yet important group i.e., individuals with physical disabilities, known to have extensive needs (e.g., accessible equipment/devices) posing a high burden on the healthcare system [68]. Another limit of the environmental scan is that we did not search for medical specialist researchers that take part in KT research on physical disabilities. We decided to target rehabilitation professions as the KT research is specifically scarce in these professions [69, 70].

The next steps consist of addressing priority strategies and engage key stakeholders to achieve our mission. The development of our strategic initiative should help build capacity in KT research in the field of rehabilitation.

## Conclusion

Valuable information was gathered on individuals conducting KT research in physical rehabilitation and on gaps and areas in need of further exploration. A strategic plan outlining priority goals with corresponding activities and strategies was produced that can be pursued to advance the rehabilitation KT research. The final consultation process with key stakeholders ensures that the plan is aligned with current and future priority areas for KT research.

Integration of new knowledge in the current health care system and advancement of KT research should be guided by a strategic orientation. The development of our strategic initiative along with the implementation of our strategic plan represent key prospects for KT science in rehabilitation for physical disabilities.

## Supplementary information

**Additional file 1.** Environmental scan eligibility criteria. Describes the eligibility criteria for the selection of researchers and projects in the environmental scan.

**Additional file 2.** Search Strategy, Criteria for Universities. Describes the search strategy for univeristies in the environmental scan.

**Additional file 3.** Search Strategy: Research Centers Criteria. Describes the search strategy for research centers in the environmental scan.

**Additional file 4.** Search Strategy, Funding Agencies and Organizations. Describes the search strategy for funding agencies and organizations in the environmental scan.

**Additional file 5.** Description of the KT-SIRQ working group members. Describes the KT-SIRQ members’ research and client population expertise.

**Additional file 6.** Expert consultation questions. Presents the five questions asked to expert for the validation process of the strategic plan.

**Additional file 7.** Projects Identified per Funding Agency or Organization. Presents results of the projects found through the environmental scan from the funding agencies and organizations.

**Additional file 8.** Goal, Strategies and Tactics Prioritization. Prensents the strategic plan developped.

## Data Availability

The datasets used and analysed during the current study are available from the corresponding author upon reasonable request.

## Abbreviations

CIHR: Canadian Institute of Health Research
EBP: Evidence-based practice
KT: Knowledge translation
KTA: Knowledge-to-action
KT-SIRQ: Knowledge Translation Strategic Initiative in Rehabilitation in Quebec
MHSS: Ministry of Health and Social Services
SPOR: Strategy for Patient-Oriented Research
