# Supplementary material for: A strategic initiative to facilitate knowledge translation research in rehabilitation
Source: BMC Health Serv Res. 2020 Oct 23;20:973. doi: 10.1186/s12913-020-05772-8 (PMC7585309; doi:10.1186/s12913-020-05772-8)
Supplement: Supplementary file 1 — Additional file 1. Environmental scan eligibility criteria. Describes the eligibility criteria for the selection of researchers and projects in the environmental scan. [file 12913_2020_5772_MOESM1_ESM.pdf]

Manuscript title: A Strategic Initiative to Facilitate Knowledge Translation Research in Rehabilitation (Montpetit-Tourangeau et al., 2020)

Additional File 1: Environmental scan eligibility criteria

Type of data	Eligibility criteria
Researchers found (universities, research centers and FRQS)	Inclusion criteria:<br>1) Researcher's description, publication or project include one of these terms<br>- Transfert<br>- Connaissances<br>- Intégration<br>- Implantation<br>- Données probantes<br>- Synthèse<br>- Guide de pratique<br>- Outil de prise de décision<br>- Algorithme<br>- Outil de connaissances<br>- Application<br>- Transfer<br>- Knowledge<br>- Translation<br>- Integration<br>- Implementation<br>- Evidence based practice<br>- Synthesis<br>- Guideline<br>- Decision making tool<br>- Algorithm<br>- Knowledge tool<br><br>2) AND has a field of work related to physical disabilities<br><br>Exclusion criteria:<br>1) A person not eligible to receive funding<br>2) Rehabilitation conducted in the field of mental health<br>3) Research conducted in other unrelated fields of research<br>4) Retired researcher<br>5) No identification of their work in KT in their description
Projects funded by all funding agencies and organizations found	Inclusion criteria:<br>1) Project's description or title include one of these terms:<br>- Transfert<br>- Connaissances<br>- Intégration,<br>- Données probantes<br>- Implantation<br>- Optimiser<br>- Application<br>- Evaluation<br>- Knowledge<br>- Integration<br>- Implementation<br>- Evidence based practice<br>- Dissemination<br>- Guide<br>-Practice guideline<br>- Optimize<br><br>2) Description rely to KT<br><br>3) Field of work related to physical disabilities<br><br>Exclusion criteria:<br>1) A person not eligible to receive funding<br>2) Rehabilitation conducted in the field of mental health<br>3) Research conducted in other unrelated fields of research
