# Supplementary material for: A strategic initiative to facilitate knowledge translation research in rehabilitation
Source: BMC Health Serv Res. 2020 Oct 23;20:973. doi: 10.1186/s12913-020-05772-8 (PMC7585309; doi:10.1186/s12913-020-05772-8)
Supplement: Supplementary file 2 — Additional file 2. Search Strategy, Criteria for Universities. Describes the search strategy for univeristies in the environmental scan. [file 12913_2020_5772_MOESM2_ESM.pdf]

Manuscript title: A Strategic Initiative to Facilitate Knowledge Translation Research in Rehabilitation (Montpetit-Tourangeau et al., 2020)

Additional File 2: Search Strategy, Criteria for Universities

**Universities**	**Schools or departments**	**Consultation date**
Université de Montréal	École de réadaptation<br>École d'orthophonie et audiologie<br>Faculté des sciences infirmières<br>Département de psychologie<br>Département de kinésiologie	Between June and July 2015
Université McGill	School of Rehabilitation<br>School of Nursing<br>Kinesiology and physical education<br>School of communication sciences and disorders<br>Department of Psychology	
Université Sherbrooke	Faculté de médecine et des sciences de la santé<br>Faculté des sciences de l'activité physique<br>Département de psychologie	
Université Laval	Département de réadaptation<br>Département de kinésiologie<br>Faculté des sciences infirmières<br>École de psychologie	
Université du Québec à Trois-Rivières	Département de chiropratique<br>Département d'ergothérapie<br>Département d'orthophonie<br>Département de psychologie<br>Département des sciences de l'activité physique<br>Département des sciences infirmières	
Concordia University	All programs and departments by key words	
Université du Québec à Montréal	All programs and departments by key word	
Université du Québec à Chicoutimi	All departments	
