# Supplementary material for: A strategic initiative to facilitate knowledge translation research in rehabilitation
Source: BMC Health Serv Res. 2020 Oct 23;20:973. doi: 10.1186/s12913-020-05772-8 (PMC7585309; doi:10.1186/s12913-020-05772-8)
Supplement: Supplementary file 3 — Additional file 3. Search Strategy: Research Centers Criteria. Describes the search strategy for research centers in the environmental scan. [file 12913_2020_5772_MOESM3_ESM.pdf]

Additional File 3: Search Strategy: Research Centers Criteria

**Research centers**	**Axes of research**
Center for Interdisciplinary Research in Rehabilitation of Greater Montreal [69]	All full and associate members
Centre de recherche de l'Institut universitaire de gériatrie de Montréal (CRIUGM)	All researchers (laboratory directors) and associate researchers
Centre de recherche du CHU Sainte-Justine	Brain and Child Development (Topics: Neurodevelopmental Studies),<br>Metabolic Health and Complex Diseases (Topics: Genetic and metabolic diseases in Quebec: diagnosis, mechanisms and interventions, Cardiometabolic and vascular health: genetic and environmental approaches, Respiratory health),<br>Musculoskeletal Diseases and Rehabilitation (all topics)
Center for Interdisciplinary Research in Rehabilitation and Social Integration (CIRRIS)	All researchers
Research Institute of McGill University Health Centre	Regular members on Health Outcomes, Cardiovascular Diseases and Critical Care, Musculoskeletal Disorders, Neurosciences and Respiratory Health
Centre de recherche du CHUM	All researchers
Centre de recherche sur le vieillissement de Sherbrooke	All researchers
