# Supplementary material for: A strategic initiative to facilitate knowledge translation research in rehabilitation
Source: BMC Health Serv Res. 2020 Oct 23;20:973. doi: 10.1186/s12913-020-05772-8 (PMC7585309; doi:10.1186/s12913-020-05772-8)
Supplement: Supplementary file 4 — Additional file 4. Search Strategy, Funding Agencies and Organizations. Describes the search strategy for funding agencies and organizations in the environmental scan. [file 12913_2020_5772_MOESM4_ESM.pdf]

Additional File 4: Search Strategy, Funding Agencies and Organizations

**Funding agency or organization**	**Search strategy**	**Consultation date**
Fonds de recherche du Québec (FRQ)	By researcher:<br>Search by researcher name (all researchers)<br>Field of application:<br>Public Health OR<br>Foundations and Knowledge Acquisition OR<br>Health System Management<br>Key words and Research Interests<br>Connaissance OR Probante OR Implantation OR Transfert<br>AND Knowledge OR Evidence OR Implementation OR Transfert	February 25th 2015
Fonds de recherche en santé du Québec (FRQS)	By project in FRQS<br>All domains, All establishments, All programs and from 2012-2013 to 2015-2016	October 9th 2015
Institut de recherche en santé et sécurité au travail (IRSST)	Research Projects held between 2005 and 2015<br>Research domains:<br>Prévention des risques mécaniques et physiques<br>Réadaptation au travail	February 17th 2015
Canadian Institute of Health Research (CIHR)	Research Projects<br>Program: Program Family<br>Operating Grant<br>All year/month<br>Peer review Committee<br>Knowledge Translation Research	February 19th 2015
Office des personnes handicapées du Québec [70]	Research Projects<br>Financed by the OPHQ<br>All documents with subject: Santé et réadaptation	March 6th 2015
Institut national d'excellence en santé et en services sociaux (INESS)	Research Projects after 2005<br>Publications: Transfert des connaissances OR Knowledge OR Implementation OR implantation OR application	March 6th 2015
Social Sciences and Humanities Research Council of Canada	Year: Between 2005 and 2013<br>All programs and grants opportunities<br>Domain: health<br>Terms of research: Transfert OR Connaissances OR Implantation OR Application OR Implementation OR Knowledge OR transfer OR translation	March 6th 2015
Réseau provincial de recherche en adaptation-réadaptation (REPAR)	Project funded between 2005 and 2015 related to KT in rehabilitation	Between March and April 2015
Quebec Network for Research in Aging - Réseau Québécois de recherche sur le vieillissement (RQRV)		

Association des établissements de réadaptation en déficience physique du Québec (AERDPQ)		
Association québécoise d'établissements de santé et de services sociaux (AQESSS)		
Institut national de santé publique du Québec (INSPQ)		
Association francophone pour le savoir (ACFAS)		
Ordre des ergothérapeutes du Québec (OEQ)		
Ordre professionnel de la physiothérapie du Québec (OPPQ)		
Edith Strauss Foundation		March 6th 2015
