# Supplementary material for: A strategic initiative to facilitate knowledge translation research in rehabilitation
Source: BMC Health Serv Res. 2020 Oct 23;20:973. doi: 10.1186/s12913-020-05772-8 (PMC7585309; doi:10.1186/s12913-020-05772-8)
Supplement: Supplementary file 5 — Additional file 5. Description of the KT-SIRQ working group members. Describes the KT-SIRQ members’ research and client population expertise. [file 12913_2020_5772_MOESM5_ESM.pdf]

Manuscript title: A Strategic Initiative to Facilitate Knowledge Translation Research in Rehabilitation (Montpetit-Tourangeau et al., 2020)

Additional File 5. Description of the KT-SIRQ working group members

Name	Expertise	Client population expertise
Aliki Thomas<br>PhD, OT	Evidence-based practice Implementation science<br>Use of theory in KT interventions<br>EBP education	Stroke and professional education
Dahlia Kairy<br>PhD, PT	Rehabilitation technology evaluation<br>Implementation science<br>Telerehabilitation	Adults in rehabilitation, with focus on stroke and neurological conditions
Sara Ahmed<br>PhD, PT	Health outcome<br>evaluation<br>E-health technologies<br>Evidence-based practice	Musculoskeletal, respiratory, neurological conditions
Dana Anaby<br>PhD, BOT	Participation-based interventions and measurements;<br>Implementation strategies	Children and youth with physical disabilities
André Bussières<br>PhD, DC	Evidence-based guidelines developments and uptake<br>Knowledge synthesis<br>Implementation research<br>Professional behaviour change	Musculoskeletal health
Chantal Camden,<br>PhD, PT	Integrated knowledge translation<br>Evidence-vased practice in rehabilitation<br>Evaluation research<br>Participatory research	Childhood Disabilities
Marie-Eve Lamontagne<br>PhD, OT	Knowledge translation<br>Implementation of best practices	Neurotraumatology (traumatic brain injury, spinal cord injury and stroke)
Annie Rochette<br>PhD, OT	Knowledge transfer and identification of facilitators and barriers for implementation of EBP<br>Leader of the www.strokengine.ca	Stroke
Keiko Shikako-Thomas<br>PhD, OT	KT Process between research and policymaking, Knowledge translation tools for clinicians and families	Childhood Disabilities
Katherine Montpetit-Tourangeau, PhD (candidate), MSc, PT	Implementation science<br>Development and assessment of education strategies	Musculoskeletal and professional education

BOT: Baccalaureate in Occupational Therapy

DC: Doctor of Chiropractic

MSc: Master of Science degree

OT: Occupational Therapist

PhD : Doctor of Philosophy

PT: Physical Therapist
