# Supplementary material for: A strategic initiative to facilitate knowledge translation research in rehabilitation
Source: BMC Health Serv Res. 2020 Oct 23;20:973. doi: 10.1186/s12913-020-05772-8 (PMC7585309; doi:10.1186/s12913-020-05772-8)
Supplement: Supplementary file 6 — Additional file 6. Expert consultation questions. Presents the five questions asked to expert for the validation process of the strategic plan. [file 12913_2020_5772_MOESM6_ESM.pdf]

Manuscript title: A Strategic Initiative to Facilitate Knowledge Translation Research in Rehabilitation (Montpetit-Tourangeau et al., 2020)

Additional File 6: Expert consultation questions

Do you find the summary and strategic plan coherent? Please specify.

Do you think the strategic plan elaborated is realistic? Please specify.

Do you think the strategic plan corresponds to knowledge translation needs and trends?

What would you edit or add to the strategic plan?

Do you have any other comments or suggestions for the strategic plan?
