# Supplementary material for: A strategic initiative to facilitate knowledge translation research in rehabilitation
Source: BMC Health Serv Res. 2020 Oct 23;20:973. doi: 10.1186/s12913-020-05772-8 (PMC7585309; doi:10.1186/s12913-020-05772-8)
Supplement: Supplementary file 7 — Additional file 7. Projects Identified per Funding Agency or Organization. Presents results of the projects found through the environmental scan from the funding agencies and organizations. [file 12913_2020_5772_MOESM7_ESM.pdf]

Additional File 7: Projects Identified per Funding Agency or Organization

Funding agency or organization	Information found
Fonds de recherche du Québec (FRQ)	14 projects related to KT and physical disabilities
Institut de recherche en santé et sécurité au travail (IRSST)	4 projects related to KT and physical disabilities
Canadian Institute of Health Research (CIHR)	5 projects related to KT and physical disabilities
Office des personnes handicapées du Québec [70]	No project found
Institut national d'excellence en santé et en services sociaux (INESS)	2 projects related to KT and physical disabilities
Social Sciences and Humanities Research Council of Canada	No project found
Réseau provincial de recherche en adaptation-réadaptation (REPAR)	20.5 projects related to KT and physical disabilities (one project funded half by the REPAR and half by the OPPQ)
Quebec Network for Research in Aging - Réseau Québécois de recherche sur le vieillissement (RQRV)	No project found
Association des établissements de réadaptation en déficience physique du Québec (AERDPQ) with the Association québécoise d'établissements de santé et de services sociaux (AQESSS)	5 projects related to KT and physical disabilities
Institut national de santé publique du Québec (INSPQ)	No project found
Association francophone pour le savoir (ACFAS)	No project found
Ordre des ergothérapeutes du Québec (OEQ)	No project found
Ordre professionnel de la physiothérapie du Québec (OPPQ)	1.5 projects related to KT and physical disabilities (one project funded half by the REPAR and half by the OPPQ)
Edith Strauss Foundation	42 projects related to KT with physical disabilities
