# Supplementary material for: A strategic initiative to facilitate knowledge translation research in rehabilitation
Source: BMC Health Serv Res. 2020 Oct 23;20:973. doi: 10.1186/s12913-020-05772-8 (PMC7585309; doi:10.1186/s12913-020-05772-8)
Supplement: Supplementary file 8 — Additional file 8. Goal, Strategies and Tactics Prioritization. Prensents the strategic plan developped. [file 12913_2020_5772_MOESM8_ESM.pdf]

Additional File 8: Goal, Strategies and Tactics Prioritization

(Delphi consensus)

Vision: To enhance the health of individuals with physical disabilities in Quebec by advancing KT research in rehabilitation science.
Mission: To build capacity and promote collaborative KT research in rehabilitation to improve the delivery of services and ultimately the health and wellbeing of individuals with physical disabilities.
The goals are to:
1) Build capacity in KT research and implementation and promote networking
2) Identify and engage relevant stakeholders (e.g., consumers of rehabilitation services, service providers, organizations, decision makers) to support the mission of the Quebec KT Rehabilitation Strategic Initiative
3) Be a catalyst for the creation, application and evaluation of innovative and effective KT strategies for individuals with physical disabilities
4) Advance KT research

GOALS	STRATEGIES	TACTICS/ACTIONS
**1. BUILDING CAPACITY Build capacity in KT research and implementation and promote networking**	A. Increase access to knowledge in KT	a) Conduct virtual journal club in KT
		b) Provide expertise and resources to stakeholders (including to identify knowledge synthesis gaps, access to effective KT strategies
		c) Offer end of grants, integrated KT workshops (grant writing)
		d) Promote the sharing of KT information
		e) Create repository of KT tools (indicators, frameworks, references, measures, presenting examples of effective and ineffective KT strategies, open access to evaluation tools)
		f) Offer virtual training program (workshop for managers and clinicians)
		g) Advertise about KT conferences and Café scientifique
		h) Integrate monitoring of ongoing literature (watch)
		i) Promote continuing professional development (KT Canada National Series)
		j) Mentor young KT investigators and incorporate grad students in KT meetings
		a) Increase awareness of networking and conferences, encourage joining existing communities of practice, advertise KT program and participate to career days

	B. Promoting KT research and implementation	b) Promote continuing professional development (KT Canada National Series, KT courses, workshops, forums, conferences, fellowships, training opportunities, social media)
		c) Increase the value and the awareness in KT
		d) Reach out to other schools and departments (identify relevant training program already existing and influence the KT content)
		e) Map joint workshops (multidisciplinary)
		f) Identify and advocate for knowledge brokers (champions, changing agent)
	C. Influence funding opportunities	a) Engage with funding agencies and organizations
		b) Encourage researchers to incorporate trainees' funding to the requested budget to support trainees and offer researchers templates on allocating budget in KT for grants
		c) Map funding opportunities including Patient scholarships
**2. ENGAGE STAKEHOLDERS Identify and engage relevant stakeholders (e.g., consumers of rehabilitation services, service providers, organizations, decision makers) to support the mission of the Qc KT Rehab Strat Initiative**	A. Identify and engage relevant stakeholders and their needs and priorities	a) Conduct and/or access existing environmental scan (researchers, funding agencies, organizations (directors of rehab programs or centers, program managers, clinicians, professional associations, academic institutions, quality improvement and evaluation managers or directors)
		b) Identify consumers of rehabilitation services (example: patient engagement group)
		c) Conduct and or access existing assessments, focus groups, advisory committee, …)
		d) Provide incentives to participate (compensation for their participation, parking…)
		e) Hold annual meeting
		f) Contact stakeholders to identify key players within respective organization
	B. Create a sustainability plan for KT-SIRQ	a) Create sub-committee to approach stakeholders for funding
		b) Maintain interest by providing biannual reports and feedback (showing output, outcomes)
		c) Expand network
**3. CATALYST FOR KT Be a catalyst for the creation, application and evaluation of innovative and effective KT for individuals with physical disabilities**	A. Support the continuous implementation of evaluation and feedback of interventions and outcomes	a) Support the uptake of effective KT strategies
		b) Support organizations in accessing high quality, reliable evidence (repository)
		c) Promote the use of standardized evaluation frameworks to guide program evaluation
		d) Support the integration of KT approaches as part of program evaluation (QI managers)
		e) Influence organizations' uptake of common indicators (example: electronic health records and administration data base)

	B. Promote sustainability of practice change	a) Help organizations identify champions to ensure sustainability of practice changes
		b) Promote ongoing involvement of stakeholders (including consumers) as advocate for change
		c) Promote the use of feedback mechanism regarding successful outcomes to stakeholders within respective organizations
**4. Advance KT research in rehabilitation**	A. Identify gaps in KT research in rehabilitation	a) Explore the literature to identify KT research gaps (example: patient health outcomes, economic outcomes, process and professional outcomes, sustainability)
	B. Promote the use of rigorous methods in KT research in rehabilitation	a) Promote use of framework and theories in KT research (Highlight the importance of the use of theories, link with existing resources, provide tools to facilitate the selection of KT theories)
		b) Facilitate the development of complex KT interventions
		c) Promote the use of evaluation of patient outcomes
		d) Influence curriculum development (e.g., advanced KT courses)
		e) Encourage participation in research networks (e.g., communities of practice)
		f) Critically appraised topic (CAT) on a), b) and c)
